# Cardiovascular-Related Outcomes in U.S. Adults Exposed to Lead

**DOI:** 10.3390/ijerph15040759

**Published:** 2018-04-15

**Authors:** Emmanuel Obeng-Gyasi, Rodrigo X. Armijos, M. Margaret Weigel, Gabriel M. Filippelli, M. Aaron Sayegh

**Affiliations:** 1 Department of Built Environment, North Carolina Agricultural and Technical State University, Greensboro, NC 27411, USA; 2 Department of Environmental and Occupational Health, Indiana University School of Public Health, Bloomington, IN 47405, USA; rarmijos@iu.edu (R.X.A.); weigelm@iu.edu (M.M.W.); 3 Department of Earth Sciences, Indiana University Purdue University Indianapolis, Indianapolis, IN 46202, USA; gfilippe@iupui.edu; 4 Department of Epidemiology and Biostatistics, Indiana University School of Public Health, Bloomington, IN 47405, USA; msayegh@indiana.edu

**Keywords:** cardiovascular, lead exposure, occupational, clinical markers, blood pressure, lipid profile

## Abstract

Cardiovascular-related clinical markers were evaluated in this cross-sectional study of United States adults (aged ≥ 20) exposed to lead via the National Health and Nutrition Examination Survey 2007–2008 and the 2009–2010 datasets. In four quartiles of exposure—0–2 μg/dL, 2–5 μg/dL, 5–10 μg/dL, and 10 μg/dL and over, clinical and anthropometric markers were evaluated—to examine how the markers manifested in the quartiles. Associations were determined via linear regression. Finally, clinical makers, and how they manifested between exposed and less-exposed occupations, were explored in addition to how duration of exposure altered these clinical markers. In regression analysis, Diastolic Blood Pressure (DBP) and high-density lipoprotein (HDL) cholesterol, were significantly associated with blood lead level (BLL). In the occupational analysis, Systolic Blood Pressure (SBP), DBP, C-reactive protein (CRP), triglycerides, low-density lipoprotein (LDL) cholesterol, high-density lipoprotein (HDL) cholesterol, showed differences between populations in the exposed and less-exposed occupations. Regarding Agriculture, Forestry & Fishing, the duration of exposure altered SBP, CRP, and LDL cholesterol. With mining, the duration of exposure altered SBP, DBP, triglycerides, and HDL cholesterol, whereas in construction, the duration in occupation altered SBP, triglycerides, and CRP. In conclusion, lead exposure has a profound effect on the cardiovascular system, with potentially adverse outcomes existing at all exposure levels.

## 1. Introduction

Cardiovascular diseases are the leading cause of mortality both in the United States and around the world [1]. Exposure to lead potentially accelerates the development of several cardiovascular diseases or disorders such as coronary heart disease, peripheral arterial disease, left ventricular hypertrophy, and cardiac arrhythmias in acute and chronic exposure [2,3,4,5]. Indeed, lead is a risk factor for hypertension in adults, and a positive association of lead exposure with elevated blood pressure has also been identified in the literature [3,4].

Lead exposure can be either acute or chronic. Whereas acute lead exposure occurs over a short period and can be of any dose, chronic exposure occurs over a longer period of time and can also be of any dose. Many epidemiologic studies have shown an association between chronic low-level lead exposure and hypertension [6,7]. Navas-Acien and co-authors, in a systemic review of lead exposure, identified a link between the effects of chronic low-level lead exposure and cardiovascular health [3]. The authors pointed out, in a review studies involving animals, that, chronic exposure to low lead levels resulted in arterial hypertension that persists long after the cessation of lead exposure. Indeed studies have found that a 2-fold increase in BLLs (i.e., 5 to 10 ug/dL) increases systolic blood pressure (SBP) between 0.6 and 1.25 mmHg [3]. Hertz-Picciotto and co-authors also found that lead exposure is strongly associated with a causal increase SBP, DBP, and hypertension [8]. Other studies such as that by Schwartz in a meta-analysis also bolster the above-mentioned finding by discovering a strong causal association in BLLs and an elevation of SBP in men [9]. In another meta-analysis, Nawrot and co-authors found an association, albeit a weak one, between elevated SBP, DBP, and BLLs [10]. According to the review findings, even though the mechanism behind lead exposure and hypertension is still under investigation, it is speculated that such a mechanism may be related to the kidney and glomerular filtration rate, oxidative stress, lead’s effects on the renin-angiotensin-aldosterone system, nitric oxide, or soluble guanylate cyclase. Indeed according to Babiker and co-authors in a study of rats, lead exposure induces oxidative stress, apoptosis in addition to promoting ischemic-reperfusion injury [11].

The role lead plays in systemic inflammation as measured by the biomarker CRP has been varied in the literature. Khan and co-authors in an occupational study found a significant association between lead exposure and CRP [12]. In contrast, a large population-based study that examined 9145 individuals who were ≥40 years of age from the National Health and Nutrition Examination Survey 1999–2004 did not find a dose-dependent relationship between BLL and CRP after adjusting for a broad array of potential confounders leading the researchers to conclude that inflammation did not appear to be an important mediator of lead toxicity [13].

Lead may play a role in altering the enzymes involved in cholesterol synthesis. This has been manifested in the literature with a study by Kojima and co-authors which demonstrated that lead nitrate-mediated induction of hepatic hypercholesterolemia involved the activation of cholesterol biosynthetic enzymes such as 3-hydroxy3methyglutaryl-CoA reductase, farnesyl diphosphate synthase, squalene synthase, and lanosterol 14a-demethylase CYP51, a vital enzyme for cholesterol biosynthesis, in addition to the concurrent suppression of cholesterol-catabolic enzymes such as 7α-hydroxylase [14].

A study by Kristal-Boneh and co-authors [15] looking at BLL, serum total cholesterol, and other cardiovascular-related outcomes in 56 lead-exposed male industrial employees compared with 87 unexposed employees sought to ascertain potential associations between BLL and serum cholesterol in subjects occupationally exposed to lead. The researchers found that mean BLLs were 42.3 (+/−14.9) µg/dL in the exposed workers and 2.7 (+/−3.6) µg/dL in the less-exposed with the exposed subjects having elevated mean levels of total cholesterol. Ademuyiwa and co-authors [16] investigated the effects of lead exposure on risk of cardiovascular disease in those occupationally exposed as compared to those non-exposed in Abeokuta, Nigeria. In the study, they found that increased risk of cardiovascular disease was observed in the occupationally exposed because total cholesterol in artisans was between 1.5 and 2.0 times higher than in controls. In the same study they found no significant difference in the mean concentration of triglycerides in occupationally lead-exposed workers as compared to controls [16]. For their part, Kristal-Boneh and co-authors also found no significant difference between the mean triglycerides of those occupationally exposed to lead as compared to the less-exposed [15]. Our study sought to examine the effects of lead on the cardiovascular system in the US general adult population by looking at cardiovascular-related clinical markers of interest and how they manifested in exposed adults.

## 2. Materials and Methods

### 2.1. Hypothesis

In this study it was hypothesized that exposure to lead adversely affects cardiovascular function via adversely affecting blood pressure, inflammation, and lipid profiles in the study participants. In testing the above hypothesis the following objective was put forward, namely:

To investigate the effects of lead exposure on the studied participants by analyzing their SBP, DBP, CRP, triglycerides, total cholesterol, LDL cholesterol, and HDL cholesterol levels.

The analysis of BLLs and clinical markers within a sample of United States adults determined the extent to which exposure to lead potentially altered these markers. Lead’s impact on occupation was explored to determine its effects on the clinical makers of interest among those occupationally exposed to lead. Potential mechanisms of disease pathology were hypothesized as follows: exposure to lead brings about inflammation which through various intermediary steps, eventually leads to elevation of blood pressure and alterations in lipid metabolism. The sociodemographic, behavioral and anthropometric covariates made it possible to statistically control for factors associated with adverse cardiovascular outcomes. It also made it possible to make estimations about the contribution of lead to studied participants’ cardiovascular-related markers. In all, it was hypothesized that being exposed to lead would be associated with elevated SBP, DBP, CRP, triglycerides, and cholesterol (LDL and Total) with a reduction in HDL Cholesterol.

### 2.2. Research Design

Data from NHANES 2007–2010 were used to examine the association between lead and cardiovascular-related markers—SBP, DBP, CRP, total cholesterol, LDL cholesterol, HDL cholesterol, and triglycerides—in the general United States population. The 2007–2008 and the 2009–2010 data sets were pooled together using NHANES web tutorial [17]. The NHANES 2007–2010 survey was conducted by the CDC using a representative sample of the U.S. noninstitutionalized civilian population. Altogether, 12,153 adult subjects ≥20 years were included in this complex multistage, stratified cluster survey in 2007 through 2010 representing 217,057,187 people. Of the 12,153 participants, blood lead was measured in 9781 adult subjects representing an estimated 182,052,299 people. Blood pressure value levels were measured in 10,316 adult subjects, which represented an estimated 192,473,335 people in the population. CRP values levels were measured in 11,071 adult subjects which represented an estimated 205,722,599 people in the population. Triglycerides was measured for 5375, which represented an estimated 215,374,827 people. Regarding total cholesterol, value levels measured were 11,028, representing an estimated 204,918,352 people, whereas HDL cholesterol data was measured for 11,028 representing 204,918,352 people. For LDL, cholesterol values were measured for 5263 people which represented 211,686,405 people.

The biochemistry biomarkers were measured using a Beckman Synchron LX20, Beckman UniCel^®^ DxC800 Synchron at Collaborative Laboratory Services (Brea, CA, USA) and the Roche Modular P chemistry analyzer (University of Minnesota, Minneapolis, MN, USA). LDL cholesterol was calculated using the Friedewald equation. CRP was measured on a Behring Nephelometer (University of Washington, Seattle, WA, USA).

Metal assays in whole blood samples were conducted in the NHANES 2007–2010 at the Division of Laboratory Sciences, National Center for Environmental Health (NCEH) of the CDC. Blood lead was determined by inductively coupled plasma mass spectrometry (ICP-MS; CDC method No. ITB0001A).

Data management was done in accordance with the NHANES analytical guidelines relating to survey design and weighting. The software Stata SE/15.0 (StataCorp, College Station, TX, USA) was used for data management.

### 2.3. Analytical and Statistical Approaches 

This study analyzed results from adults aged 20 and older. In portions of the study, analysis was performed on those experiencing various degrees of exposure represented by BLLs in four quartiles; 0–2 µg/dL, 2–5 µg/dL, 5–10 µg/dL, 10+ µg/dL presented in this study as quartile 1, quartile 2, quartile 3, and quartile 4 respectively, which represent thresholds typically and historically used in the literature to represent elevated exposure. Association between lead and cardiovascular outcomes were explored using linear regression. Since the variables of interest were not normally distributed, natural log transformation was used for dependent and independent variables in regression analysis.

For linear regression, all independent variables were analyzed as continuous variables. The covariates of interest (gender, body mass index (BMI), ethnicity, and age), as well as “taking a prescription” for hypertension (for SBP and DBP), and consumption of alcohol (those who had taken at least 12 alcoholic drinks in the past year), smoking habits, and taking prescription medication for cholesterol for (total cholesterol, HDL, LDL, and triglycerides) were adjusted for to determine leads impact on the clinical markers of interest. Statistical analyses were performed using Stata SE/15.0, as the software allowed for adjustment for clusters and strata of the complex sample in addition to incorporating the sample weight in order to generate estimates for the total noninstitutionalized civilian population of the United States.

Occupational exposure to lead was explored by determining three occupations which had the highest and lowest BLLs at the occupation of longest duration (the occupation they had spent most of their career in). This was done to examine how the clinical makers manifested in high exposure occupations when compared to low exposure occupations. In addition, duration in occupation was examined for time intervals of 0–5 years, 5–10 years, and over 10 years to see how length of time at a lead exposed job may alter clinical markers of interest. A *p*-value of <0.05 was considered significant while a value of <0.10 was considered moderately significant. Excel 2016 was used to generate charts/figures.

## 3. Results

### 3.1. Age, BMI and Clinical Markers

Information on age and BMI and clinical makers by degree of exposure are presented in Table 1.

### 3.2. Association of BLL with Clinical Markers of Interest of All Adults

Associations of BLL were examined with each of the cardiovascular related variables (which included CRP, SBP, DBP, triglycerides, total cholesterol, LDL Cholesterol, and HDL Cholesterol variables) using separate regression models for each exposure-outcome association. All variables were adjusted for age, gender, race/ethnicity, BMI, alcohol consumption and smoking. In addition, SBP, DBP were adjusted for taking prescription medications for hypertension with triglycerides, total cholesterol, LDL, and HDL adjusted for taking medications for cholesterol. The associations of BLL and the cardiovascular-related variables are presented in Table 2 below.

### 3.3. Occupational Exposure to Lead

#### Occupations Providing Highest Exposure

**Figure 1:**
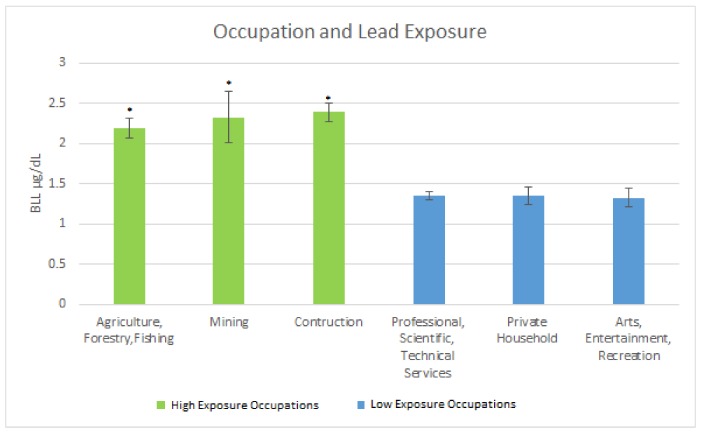
Occupation and Lead Exposure. * Significant difference between high exposure occupations as compared to low exposure occupations.

The occupations providing a high level of exposure as measured by mean BLLs were: (a) Agriculture, Forestry & Fishing 2.19 µg/dL (SE = 0.12); (b) Mining 2.33 µg/dL (SE = 0.34); and (c) Construction 2.39 µg/dL (SE = 0.12). The occupation providing the lowest levels of exposure as measured by mean BLLs were: (a) Professional, Scientific, Technical Services 1.35 µg/dL (SE = 0.05); (b) Private Household 1.35 µg/dL (SE = 0.11); and (c) Arts, Entertainment, Recreation 1.33 µg/dL (SE = 0.11). Figure 1 below illustrates the findings.

**Figure 2:**
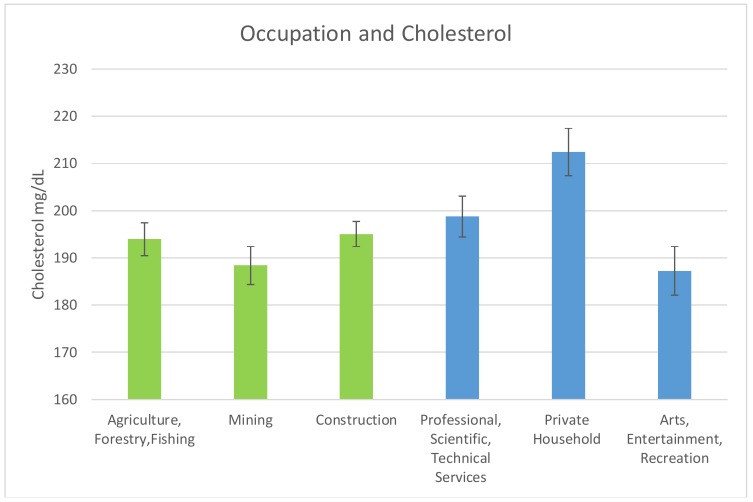
Occupation and Mean Cholesterol Levels. No statistically significant decrease in total cholesterol levels between high lead exposure occupations (Green) as compared to low lead exposure occupations (Blue).

The mean cholesterol levels in the occupations of interest are shown in Figure 2 below. For the high exposure occupations, the mean cholesterol level for Agriculture, Forestry & Fishing was 193.95 mg/dL (SE = 3.48). For Mining and Construction, the mean cholesterol levels were 188.41 mg/dL (SE = 4.02) and 195.02 mg/dL (SE = 2.66) respectively.

For the low exposure occupations, the mean cholesterol levels were: Professional Scientific, Technical Services 198.72 mg/dL (SE = 4.33); Private Household 212.42 mg/dL (SE = 5.00); and Arts Entertainment, Recreation 187.30 mg/dL (SE = 5.13).

**Figure 3:**
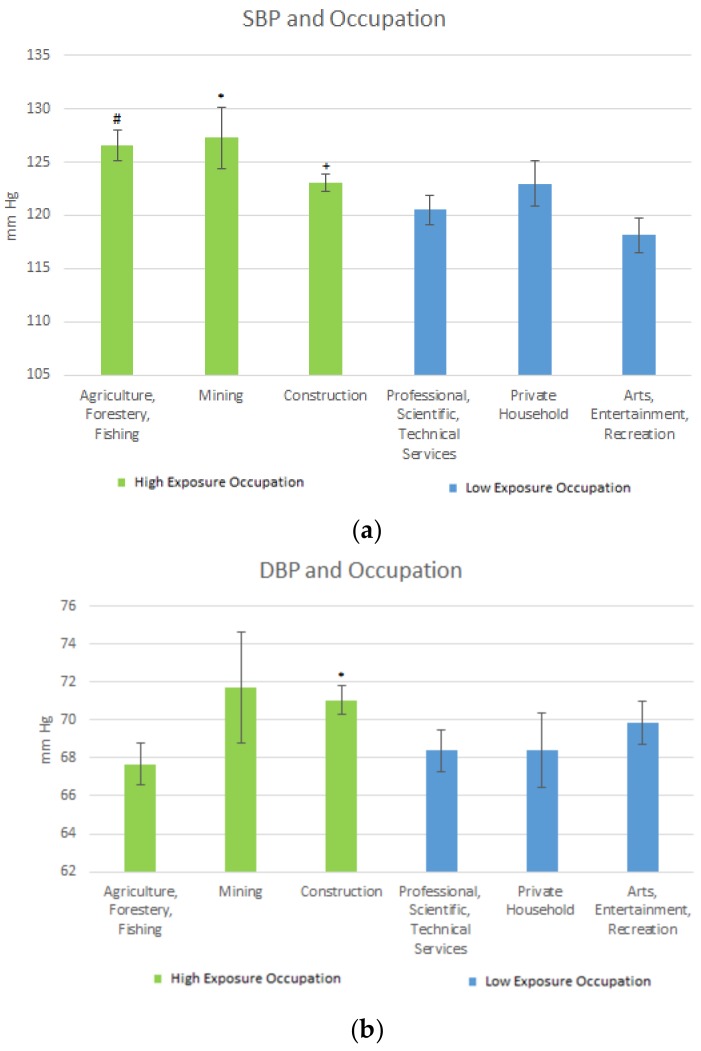
(**a**) Occupation and SBP Mean Levels. # Significant difference between Agriculture, forestry, fishing and Professional, Technical Services and Arts, Entertainment, Recreation; * Significant difference between mining and Arts, Entertainment, Recreation. Moderately for mining and Professional, Technical Service; + Significant difference between construction and Arts, Entertainment, Recreation. (**b**) Occupation and DBP Levels. * Significant difference between construction and Professional, Scientific and Technical services.

Blood pressure in these occupations was also explored. Figure 3a,b shows the SBP and DBP levels based on the longest served occupation. For the high exposure occupations, the mean SBP level regarding Agriculture, Forestry & Fishing was 126.53.58 mmHg (SE = 1.42); for Mining it was 127.26 mg/dL (SE = 2.88). For Construction, SBP level was 123.03 mmHg (SE = 0.85). For the low exposure occupations, the mean SBP levels were: Professional Scientific, Technical Services 120.51 mmHg (SE = 1.41); Private Household 122.95 mg/dL (SE = 2.15); and Arts, Entertainment, Recreation 118.15 mg/dL (SE = 1.63). See Figure 3a.

The results for DBP levels for the high exposure occupations were as follows: Agriculture, Forestry & Fishing, 67.68 mmHg (SE = 1.10); Mining, 71.72 mmHg (SE = 2.92); and Construction, 71.04 mmHg (SE = 0.77). For the low exposure occupations, the mean DBP levels were: Professional Scientific, Technical Services, 68.38 mmHg (SE = 1.10); Private Household, 68.42 mmHg (SE = 4.01); and Arts Entertainment, Recreation, 69.86 mmHg (SE = 2.99). Figure 3a,b represents the mean SBP and DBP mean levels in the longest served (duration) occupations.

**Figure 4:**
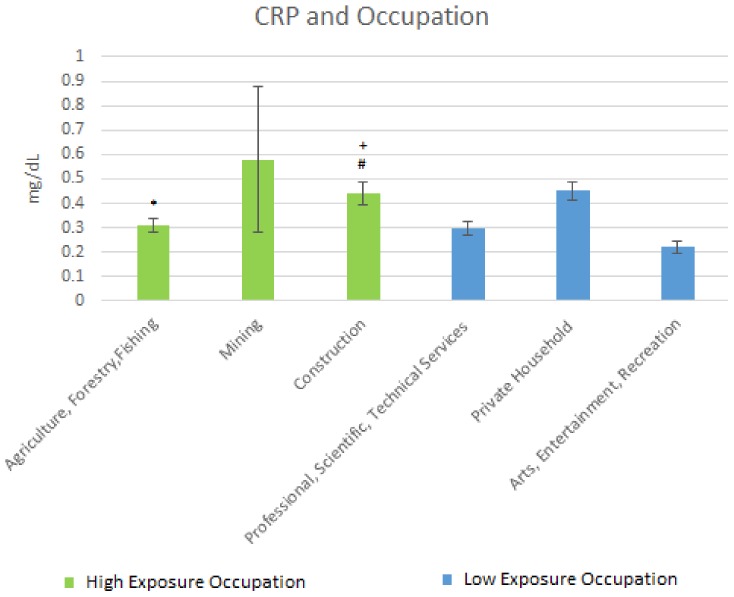
Occupation and CRP Levels. * Significant difference between Agriculture, Forestry, Fishing and Arts, Entertainment, and Recreation; + Significant difference between construction and Professional, Scientific and Technical services; # Significant difference between construction and Arts, Entertainment, and Recreation.

Results of the mean C-reactive protein by occupation are shown Figure 4 below. For the high exposure occupations, the mean CRP in Agriculture, Forestry & Fishing was 0.31 mg/dL (SE = 0.03); that for Mining was 0.58 mg/dL (SE = 0.3) whereas that for Construction was 0.44 mg/dL (SE = 0.047).

For the low exposure occupations, the mean C-reactive protein levels were: Professional, Scientific, Technical Services, 0.30 mg/dL (SE = 0.028); Private Household, 0.45 mg/dL (SE = 0.038); and Arts, Entertainment, Recreation, 0.22 mg/dL (SE = 0.024).

**Figure 5:**
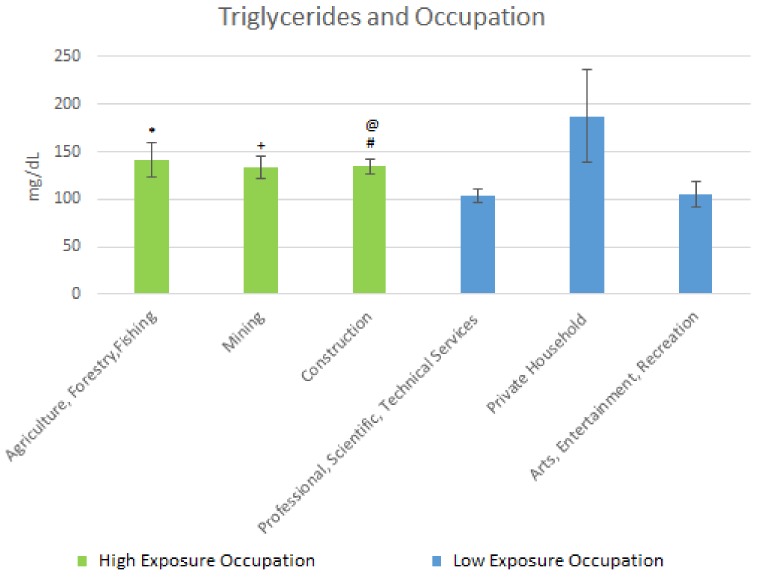
Occupation and Triglycerides Levels. * Moderately significant difference between Agriculture, Forestry, Fishing and Professional, Scientific, Technical Services; + Significant difference between Mining and Professional Scientific, Technical Services; # Significant difference between construction and Professional Scientific, Technical Services; @ Moderately significant difference between Construction and Arts, Entertainment, Recreation.

Results of the mean triglyceride levels are shown in Figure 5 below. For the high exposure occupations, the following results were obtained. The mean triglyceride level for Agriculture, Forestry & Fishing was 141.50 mg/dL (SE = 17.91). For Mining, the level was 133.50 mg/dL (SE = 11.60) and that for Construction was 134.94 mg/dL (SE = 7.79). For the low exposure occupations, the mean triglyceride level levels were: Professional, Scientific, Technical Services, 103.59 mg/dL (SE = 6.85); Private Household, 187.48 mg/dL (SE = 48.95); and Arts Entertainment, Recreation, 105.17 mg/dL (SE = 12.93).

**Figure 6:**
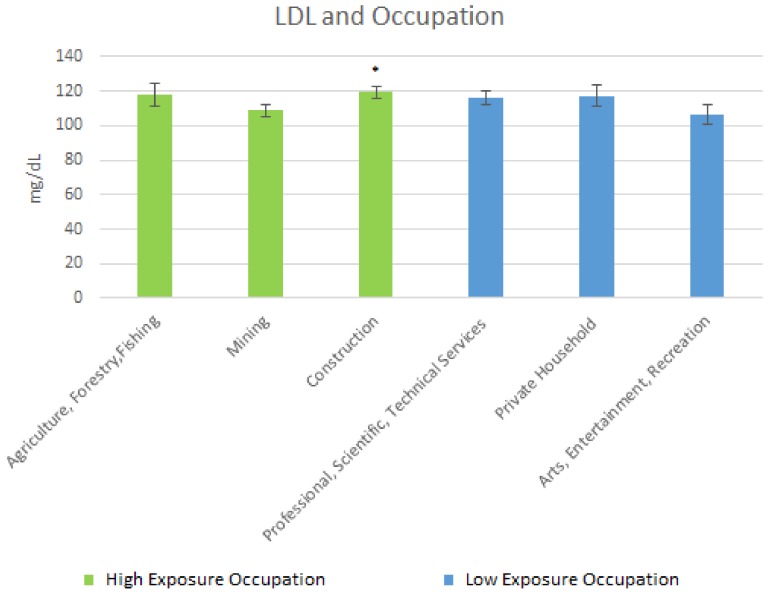
Occupation and LDL Levels. * Moderately significant difference between Construction and Arts, Entertainment, Recreation.

The mean LDL cholesterol levels are shown in Figure 6 below. For the high exposure occupations, the following cholesterol levels were obtained: Agriculture, Forestry & Fishing, 117.82 mg/dL (SE = 6.58); Mining, 108.72 mg/dL (SE = 3.89); and Construction, 119.45 mg/dL (SE = 3.45). For the low exposure occupations, the mean LDL levels were: Professional Scientific, Technical Services, 115.97 mg/dL (SE = 4.02); Private Household, 117.43 mg/dL (SE = 6.17); and Arts Entertainment & Recreation, 106.58 mg/dL (SE = 5.98).

**Figure 7:**
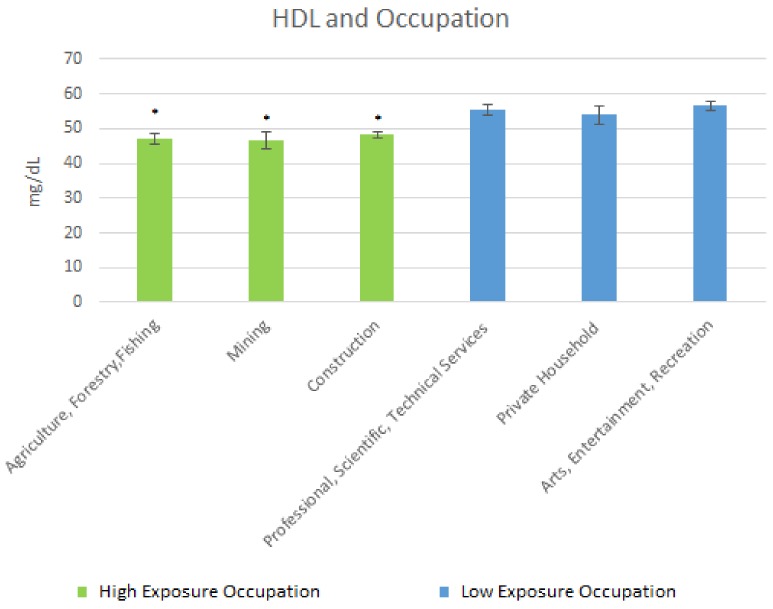
Occupation and Mean HDL Levels. * Significant difference between low exposure occupations and high exposure occupations.

The mean HDL cholesterol levels are shown in Figure 7 below. For the high exposure occupations, results of the mean HDL cholesterol levels were as follows: Agriculture, Forestry & Fishing, 46.93 mg/dL (SE = 1.51); Mining, 46.79 mg/dL (SE = 2.41); and Construction, 48.3 mg/dL (SE = 0.79). For the low exposure occupations, the mean HDL levels were: Professional, Scientific, Technical Services, 55.27 mg/dL (SE = 1.58); Private Household, 53.93 mg/dL (SE = 2.59); and Arts Entertainment & Recreation, 56.63 mg/dL (SE = 1.3).

The effects of duration of exposure on the clinical markers of interest are shown in Table 3 below. The values represent mean levels for the clinical makers of interest during that listed time interval.

## 4. Discussion

This study sought to determine the associations of cardiovascular-related markers with BLL in US adults in addition to predicting the likelihood of elevated BLL at different exposure levels. Finally it sought to understand how lead exposure manifested in high- and low- lead exposed occupations cognizant of the fact that lead exposure in adults most commonly occurs in the workplace and industries such as the construction industry have historically been a source of lead exposure among adults [18,19].

It was determined that the occupations providing the most exposure, as measured by BLLs in adults were the Construction industry; Agriculture Forestry & Fishing; and Mining. This is in line with research that has demonstrated that jobs such as those within the construction industry are potential sources of lead exposure [20]. Agriculture Forestry & Fishing, in addition to Mining also provide avenues to keep workers exposed to toxicants such as lead [21,22]. The occupations with the least exposed population were: Professional, Scientific, and Technical Services; Private Household; and Arts, Entertainment, and Recreation. These industries were examined to see their how the clinical markers of interest varied between the high exposure occupations and the lower exposure occupations.

In comparing high exposure occupations to low exposure occupations, the mean BLLs were significantly higher when comparing the three high exposure occupations to the three low exposure occupations. Even though BLL is a marker of acute exposure this potentially hints at chronic low-level lead exposure among the highly exposed occupations as the BLL levels in these occupations were below the Occupational Safety and Health Administration’s (OSHA) lead standards which require that workers be removed from lead exposure sources when BLLs are ≥50 μg/dL in the construction industry or 60 μg/dL in general industry [23].

Mean SBP was significantly elevated when comparing the Agriculture, Forestry & Fishing industries to Professional Scientific, Technical Services, and Arts, Entertainment, and Recreation. This may be due to lead contaminated soils [24] which owing to leads persistent nature may continue to expose workers in this industry.

In Mining, mean SBP was significantly elevated when compared to Arts, Entertainment, and Recreation, while it was moderately significantly elevated for mining when compared to Professional, Scientific & Technical services. Living by mining areas exposes populations to heavy metals such as lead [25], with a resulting health outcome from the exposure potentially being hypertension [3]. Studies on blood pressure in construction workers have varied, with some studies finding no association between SBP and DBP in this occupation [26], and others finding construction workers to have elevated SBP and DBP. In this study, those in the Construction industry had significantly elevated blood pressure as compared to those in the less-exposed occupations such as Education. Regarding DBP, the mean levels were significantly elevated when comparing Construction to Professional, Scientific, and Technical services.

For CRP, Agriculture, Forestry, & Fishing was significantly elevated when compared to Arts, Entertainment, & Recreation. There was also a significant elevation between Construction and Professional Scientific and Technical services, and a moderately significant difference between Construction and Arts, and Entertainment & Recreation. This potentially speaks to the degree of systemic inflammation that comes from performing these lead exposed jobs with CRP and other markers, indicating that the cardiovascular system may bear a significant portion of this inflammation. Elevated CRP has been associated with lead exposure [27] in community-based studies as well as occupational studies; thus systemic inflammation may serve as a mediator for other biological mechanisms such as oxidative stress in those exposed to lead. These two mechanisms potentially serve as the genesis of many of the cardiovascular pathologies that comes about from lead exposure.

Regarding triglycerides, there was a significant difference in the means between Mining and Professional, and Scientific & Technical Services. In Construction, there was a significant difference in the mean levels for mining when compared to Professional and Scientific & Technical Services. There was also a moderately significant difference between Agriculture and Forestry & Fishing when compared to Professional, Scientific, & Technical Services, and between Construction and Arts, and Entertainment & Recreation. Triglyceride levels may potentially be altered by occupation; with working in lead-exposed occupations seeming to worsen triglyceride levels in adults. What is unclear is the degree to which lead alters triglyceride levels.

For LDL cholesterol, there was a moderately significant elevation in those in Construction as compared to those in Arts, Entertainment, & Recreation whereas for HDL, the less-exposed occupations were significantly elevated compared to the high exposure occupations. Lead potentially promoting LDL and diminishing HDL in the occupational analysis largely supports the results of similar occupational studies [16].

Finally, in longest held occupation, a marker of long term exposure, results for Construction demonstrated that mean BLLs were significantly elevated from the 0–5-year working period when compared to the 10 plus year working period; while it was moderately elevated from the 0–5 to 5–10-year period. In Mining BLLs were significantly elevated from the 0–5-year to the 10+ year period and from the 0–5-year period to the 5–10-year period.

Mean SBP was elevated significantly from the 0–5-year working period when compared to the 10 plus year working period in Agriculture and Mining, & Construction, while it was also significantly elevated from the 0–5-year period to the 5–10-year period in Construction. DBP was significantly elevated from the 0–5-year period to the 5–10-year period in Mining. This potentially indicates that the time spent in a job in which lead exposure exists may alter blood pressure, with outcomes possibly being time-dependent. The time interval in which the more severe pathology happens is debatable and is likely related to several factors including, genetic [28] and behavioral factors. CRP was significantly elevated from the 0–5 to 5–10-year period in Agriculture and Forestry & Fishing, and moderately elevated form the 0–5 to 10+ year period in Construction, indicating that lead exposure in these occupations may promote systemic inflammation over time, which could potentially lead to cardiovascular pathology over an extended period of time.

An observation of the data also showed that Triglycerides levels were elevated from the 5–10 to 10+ year period in Mining and Construction; and also elevated from the 0–5 to 5–10-year period in Construction. LDL cholesterol was elevated from the 0–5 to the 5–10-year period, and 0–5 to 10+ year period. HDL cholesterol level was elevated from the 5–10 to 10+ year period in Mining, and moderately decreased in the 5–10 to the 10+ year period in Construction. Lead’s potential action on these clinical markers indicates more work must be done to understand its effects over longer and shorter periods of time. This will give insight into how to properly mitigate lead exposure for those exposed to different occupations, which may present different exposure conditions, and potentially induce or promote disease to manifest in different ways.

### 4.1. On Lead and Its Role in Cardiovascular Health

According to Lanphear and co-authors, low-level environmental lead exposure substantially affects cardiovascular health [29]. In the United States, owing to the legacy of lead contamination such exposure is common. Among adults in the United States, the primary avenue by which exposure to lead occurs is in the workplace [30].

With respect to BLL and cardiovascular-related markers in all adults, significant associations between BLL, DBP, and HDL cholesterol was found in this study. In examining the clinical markers by quartile of exposure, for SBP there was a significant elevation from quartile 1 to quartiles 2 and 3. This indicates a potential relationship, with higher exposure to lead increasing SBP. For DBP, there was a significant elevation from quartile 1 when compared to quartile 4. For CRP there was a significant elevation from quartile 1 to quartile 3. This indicates that systemic inflammation potentially increases with increasing dose of lead, but it should be noted that there was not a significant association in regression analysis, which may partly be due to how the data is more skewed toward the lower BLLs. Larger databases or studies with larger groups of significantly exposed individuals may yield different results. For triglycerides, there was a significant elevation from quartile 1 when compared to quartiles 2 and 4; and for total cholesterol, there was a significant elevation from quartile 1 to quartiles 2 and 3. With respect to mean LDL cholesterol level, there was a significant elevation from quartile 1 to quartiles 2 and 4, whereas for mean HDL cholesterol level, there was a significant elevation from quartile 1 and 2; quartile 4 had the lowest mean HDL cholesterol level. All of this indicates that lead potentially accelerates the formation of LDL and total cholesterol, and hinders the production of HDL, but the dosage at which that starts is unclear.

As demonstrated in the literature, lead has been associated with increases in both SBP and DBP. Research has consistently found an association between BLL and SBP and DBP with Harlan and co-authors, using data from the second National Health and Nutrition Examination Survey, to demonstrate a direct relationship between BLL and SBP and DBPs for men and women, and for white and black persons aged 12 to 74 [31]. For their part, Nawrot and co-authors found, in a study including both men and women, that two-fold increase in blood lead concentration was associated with a 1.0 mm Hg rise in the SBP (95% CI +0.5 to +1.4 mm Hg; *p* < 0.001) and with a 0.6 mm Hg increase in the DBP (95% CI +0.4 to +0.8 mm Hg; *p* < 0.001) [10]. A comparison of the results of my study with those in the literature points to the fact that the results of my study, to a large extent, bolster what obtains in the literature regarding the positive association.

The results of lead and its effects on lipoproteins have been varied. Research by Cocco and co-authors found that lead-exposed patients had decreased cholesterol levels [32]. Meanwhile, Ademuyiwa and co-authors reported a significant elevation in total cholesterol for subjects exposed to lead, compared to controls in addition to finding a strong positive association between BLL and total and LDL cholesterol, but not HDL or triglycerides [16]. Kristal-Boneh and co-authors, in a study of the association between occupational lead exposure and serum cholesterol and lipoproteins found a significant elevation in the HDL and total cholesterol levels between the less exposed and exposed, which bolster the results of this study, but their study did not find a significant elevation of LDL cholesterol between the less exposed and exposed [15]. The positive association of BLL with HDL cholesterol is paradoxical. This may be explained by the data being skewed toward lower exposure. The analysis of mean values indicated that the mean HDL cholesterol values were higher for higher exposure levels as compared to lower exposure levels, but these differences were not significant. Overall, a larger dataset may have yielded different results, and lead’s diminishing effects on HDL may be at higher doses.

In the literature, findings for C-reactive protein (CRP) have varied. As noted earlier, Khan and co-authors in an occupational study found a significant association between lead exposure and CRP [12]. On the other hand, Songdej and co-authors, in a population-based study, did not find a consistent association [13].

### 4.2. Limitations of Study

Measurement of BLLs does not indicate longer-term exposure; rather, it is indicative of recent lead exposure as well as lead that has been mobilized from bone or other tissue sources with no ability to distinguish between both. Measurement of bone lead levels, particularly tibia lead level, via K-Shell X-ray Fluorescence (KSXF) would have provided more information on length of exposure, as bone lead levels are indicative of long-term cumulative exposure to lead. Both the BLLs and bone lead levels taken together would have provided the best and most comprehensive view of the participant’s exposure [33]. In attempting to overcome the limitation of long-term exposure, the length of time at occupation was analyzed, and seeing the differences in health outcomes overtime helped to give hints on the manifestations of long term exposure.

Finally, owing to the inability to adjust for covariates in the occupational analysis, and the inability to perform regression analysis on lead exposed occupations due to inadequate data in all strata for any of exposed occupations, future work should look at larger occupational databases. This will enable us to evaluate the significances found here in adjusted models for lead-exposed occupations.

## 5. Conclusions

Lead exposure was significantly associated with adverse cardiovascular-related outcomes, with elevated exposure resulting in poorer outcomes. Looking at various degrees of exposure, exposure to lead increased the odds of adverse cardiovascular-related and inflammatory clinical markers. Lastly, occupational exposure potentially plays a role in these outcomes. The findings in this study add to the extensive research that has demonstrated that lead exposure may be an important risk factor for cardiovascular-related dysfunction in populations experiencing various degrees of exposure. Based on this study, it is suggested that a critical need exists to test novel interventions capable of mitigating and subsequently eliminating the impact of lead on cardiovascular health. Studies aimed at interventions that mitigate and/or eliminate the harmful effects of lead on the environment and on human health are still required for successful optimal health management.

## Figures and Tables

**Table 1:** Clinical Factors and Quartiles of Exposure.

Variables	Quartile 1 (0–2 µg/dL)	Quartile 2 (2–5 µg/dL)	Quartile 3 (5–10 µg/dL)	Quartile 4 (10+ µg/dL)
Age *	44.25 (0.32)	56.05 (0.54)	54.77 (1.13)	47.56 (2.56)
BMI	25.79 (0.17)	25.91 (0.18)	26.06 (0.49)	26.40 (0.92)
BLL	1.09 (0.01)	2.78 (0.02)	6.40 (0.10)	16.11 (1.40)
SBP **	119.56 (0.31)	126.39 (0.57)	127.75 (1.71)	126.37 (5.09)
DBP ***	69.9 (0.38)	70.54 (0.40)	70.07 (0.87)	76.36 (1.75)
CRP +	0.38 (0.01)	0.41 (0.02)	0.46 (0.07)	0.26 (0.04)
Triglycerides ++	128.41 (2.16)	141.69 (4.11)	135.61 (10.41)	184.64 (20.72)
Total Cholesterol +++	194.75 (0.67)	203.58 (0.89)	202.28 (3.25)	203.39 (6.78)
LDL Cholesterol #	114.67 (0.82)	121.67 (1.18)	118.02(4.59)	128.05 (6.29)
HDL Cholesterol ##	52.03 (0.34)	53.47 (0.51)	51.79 (1.18)	50.19 (4.16)

* *p* < 0.05 Significant difference between quartile 1 and 2, 3.** *p* < 0.05 significant difference between quartile 1, when compared to 2, 3; *** *p* < 0.05 significant difference between quartile 1 when compared to 4; + *p* < 0.05 significant difference between quartile 1 and quartile 3; ++ *p* < 0.05 significant difference between quartile 1 and 2, 4; +++ *p* < 0.05 significant difference between quartile 1 and 2, 3; # *p* < 0.05 significant difference between quartile 1 and 2, 4; ## *p* < 0.05 significant positive between quartile 1 and 2.

**Table 2:** Associations of BLL with Cardiovascular-Related Markers of Interest.

Variables	lnBPb Unadjusted (95% CI)	*p* Value	lnBPb Adjusted (95% CI)	*p* Value
CRP	−0.003 (−0.015, 0.008)	0.555	−0.011 (−0.025, 0.004) ^	0.155
SBP	1.114 (0.996, 1.233)	0.0001	0.052 (−0.233, 0.329) ^^	0.699
DBP	0.174 (0.057, 0.291)	0.005	0.268 (0.079, 0.458) ^^	0.007
Triglycerides	0.110 (0.060, 0.159)	0.0001	−0.05 (−0.173, 0.071) ^^^	0.398
Total Cholesterol	0.350 (0.283, 0.416)	0.0001	0.190 (−0.055, 0.434) ^^^	0.124
LDL Cholesterol	0.203 (0.144, 0.261)	0.0001	0.160 (−0.111, 0.431) ^^^	0.239
HDL Cholesterol	0.024 (−0.027, 0.075)	0.344	0.218 (0.060, 0.375) ^^^	0.008

^ Adjusted for age, gender, race/ethnicity, BMI, alcohol consumption and smoking; ^^ Adjusted for age, gender, race/ethnicity, BMI and those taking prescription medicines for hypertension; ^^^ Adjusted for age, gender, race/ethnicity, BMI those told to take prescription medications for cholesterol, alcohol consumption, and smoking.

**Table 3:** Longevity in occupation and health outcomes.

Time Interval	Occupation	BLL (SE)	SBP (SE)	DBP (SE)	CRP (SE)	Triglycerides (SE)	Total Cholesterol (SE)	LDL (SE)	HDL (SE)
0–5 years	Agriculture	1.72 (0.38)	120.50 (3.03)	62.92 (4.82)	0.20 (0.04)	147.48 (46.42)	177.00 (6.59)	85.79 (8.79)	48.66 (3.00)
5–10 years	Agriculture	2.18 (0.48)	121.38 (3.97)	67.15 (2.05)	0.47 (0.22) ^+^	139.39 (21.29)	196.03 (7.81)	124.25 (7.25)	52.01 (4.87)
10+ years	Agriculture	2.25 (0.12)	127.92 (1.73) ^@^	68.34 (1.18)	0.30 (0.04)	146.28 (15.93) ^@^	195.95 (4.61)	123.30 (8.51)	46.00 (1.55)
0–5 years	Mining	1.05 (0.43)	116.94 (1.64)	64.82 (4.90)	0.41 (0.17)	145.27 (42.41)	171.60 (14.76)	111.12 (7.74)	47.87 (3.61)
5–10 years	Mining	1.61 (0.28)	128.01 (9.48)	81.40 (4.93)	0.27 (0.07)	97.58 (19.15)	190.34 (7.53)	114.41 (1.01)	35.46 (5.65)
10+ years	Mining	2.57 (0.38) ^+,@^	127.96 (2.23) ^@^	69.54 (4.43)	0.68 (0.39)	144.43 (14,71) *	189.38 (4.84)	107.47 (5.00)	49.78 (2.76) *
0–5 years	Construction	1.58 (0.76)	119.67 (2.21)	69.38 (2.09)	0.29 (0.06)	110.98 (10.27)	191.74 (6.66)	118.00 (8.11)	48.45 (2.06)
5–10 years	Construction	2.71 (0.69)	116.43 (1.16)	69.20 (1.62)	0.35 (0.05)	158.19 (27.61) ^+^	200.83 (5.42)	119.71 (5.58)	50.02 (2.52)
10+ years	Construction	2.53 (0.24) ^@,M2^	125.52 (1.10) ^+,@^	71.93 (0.90) ^M2^	0.50 (0.07) ^M1^	143.31 (7.76) *	194.59 (2.82)	119.97(4.23)	47.89 (1.12) ^M2^

^@^ Significantly different from 0–5 years to 10+; * Significantly different 5–10 years to 10+; ^+^ Significantly difference 0–5 to 5–10; ^M1^ moderately significant difference between 0–5 years and 10+ years; ^M2^ moderately significant difference between 5–10 years and 10+ years.
